# Fine Mapping of a Major Pleiotropic QTL Associated with Sesamin and Sesamolin Variation in Sesame (*Sesamum indicum* L.)

**DOI:** 10.3390/plants10071343

**Published:** 2021-06-30

**Authors:** Fangtao Xu, Rong Zhou, Senouwa Segla Koffi Dossou, Shengnan Song, Linhai Wang

**Affiliations:** Key Laboratory of Biology and Genetic Improvement of Oil Crops, Ministry of Agriculture and Rural Affairs, Oil Crops Research Institute of the Chinese Academy of Agricultural Sciences, No. 2 Xudong 2nd Road, Wuhan 430062, China; xufangtao521@163.com (F.X.); rongzzzzzz@126.com (R.Z.); dossouf@yahoo.fr (S.S.K.D.); songshengnan1988@163.com (S.S.)

**Keywords:** sesame, QTL mapping, pleiostropic locus, sesamin and sesamolin, candidate gene

## Abstract

Deciphering the genetic basis of quantitative agronomic traits is a prerequisite for their improvement. Herein, we identified loci governing the main sesame lignans, sesamin and sesamolin variation in a recombinant inbred lines (RILs, F8) population under two environments. The content of the two lignans in the seeds was investigated by HPLC. The sesamin and sesamolin contents ranged from 0.33 to 7.52 mg/g and 0.36 to 2.70 mg/g, respectively. In total, we revealed 26 QTLs on a linkage map comprising 424 SSR markers, including 16 and 10 loci associated with sesamin and sesamolin variation, respectively. Among them, *qSmin_11.1* and *qSmol_11.1* detected in both the two environments explained 67.69% and 46.05% of the phenotypic variation of sesamin and sesamolin, respectively. Notably, *qSmin11-1* and *qSmol11-1* were located in the same interval of 127–127.21 cM on LG11 between markers ZMM1776 and ZM918 and acted as a pleiotropic locus. Furthermore, two potential candidate genes (*SIN_1005755* and *SIN_1005756*) at the same locus were identified based on comparative transcriptome analysis. Our results suggest the existence of a single gene of large effect that controls expression, both of sesamin and sesamolin, and provide genetic information for further investigation of the regulation of lignan biosynthesis in sesame.

## 1. Introduction

Sesame is a worldwide oilseed crop that, owing to its nutritional and therapeutic qualities, has gained substantial attention [1]. Its seeds are rich in oil, proteins, vitamins, minerals, and a class of lignans highly sought after by humans because of their various biological properties [2,3]. In some places, the daily intake of lignans by males and females from sesame seeds and oil was estimated at 18.39 and 13.26 mg/person, respectively [4]. Lignans are chemically classified as monolignol dimers [5]. Sesame seeds and products contain a large number of lignans, among which sesamin and sesamolin are the major ones [6]. Many studies had reported various pharmacological abilities of sesamin and sesamolin, including anti-inflammatory, anti-oxidative, anti-cancerogenic, anti-hypertensive, anti-proliferative, anti-melanogenesis, auditory-protective, anti-cholesterol and anti-aging [7,8,9,10]. The total lignan content is a critical factor in sesame seed quality evaluation [11].

The health-promoting properties of sesamin and sesamolin have expanded the demand for sesame products containing high lignans [12]. Thus, breeding for high oil, lignans contents and seed yield is the main objective of sesame breeders. Dissecting the genetic basis and understanding the genetic control of lignan biosynthesis is a prerequisite for achieving the target. Sesamin and sesamolin contents in sesame seed are quantitative polygenic traits controlled by additive and dominance effects [13,14,15]. The sesamin and sesamolin contents vary broadly in sesame germplasms [16,17,18]. Their contents are influenced by other seed components, including oil, protein, and lignin. A significant positive correlation between the content of oil and lignans and a negative correlation between the content of protein and oil and protein and lignans have been detected [19,20,21,22]. The same correlations have also been found in an RIL population between seed oil, protein, and sesamin contents by Wu et al. [23]. In addition to seed components, differences in the content of seed lignans can also be correlated with the seed color and growing conditions [16,18,24].

Sesamin and sesamolin biosynthesis occur in sesame in the general phenylpropanoid pathway from phenylalanine and tyrosine [25]. Ono et al. [26] discovered that a cytochrome P450 gene (*CYP81Q1*) named piperitol/sesamin synthase (PSS) is responsible for sesamin biosynthesizing from pinoresinol through piperitol by the formation of two methylenedioxy bridges. The biosynthesis of pinoresinol, the basal lignan from two aciral E-coniferyl alcohols, is regulated by a dirigent protein [2,5]. *CYP92B14*, another cytochrome P450 gene, was discovered recently to be responsible for sesamolin and sesaminol biosynthesis from sesamin [27]. Functional studies showed that *CYP81Q1* and *CYP92B14* operate coordinately in sesame developing seeds [27].

Since the sesame genome (Zhongzhi No. 13) was published [28], genetic maps harboring SSR markers and RAD tags have been constructed [29,30], and a list of QTLs and candidate genes have been figured out, including those that control oil biosynthesis [12,31]. QTL mapping is a useful approach in genetics to detect loci associated with complex quantitative traits in crops [32]. It has been used to reveal the genetic basis of various agronomic traits in sesame [30,33,34,35]. However, apart from the genes involving in biosynthesis, only eight SNPs and five QTLs for sesamin and sesamolin content variations were detected [23,28,36]. No candidate causative gene was identified, and the regulation of sesamin and sesamolin biosynthesis in sesame developing seeds still needs to be clarified. Hence, this study was undertaken to screen the variability of sesamin and sesamolin content available in an RIL population of sesame that was grown in two environments. The loci governing sesamin and sesamolin content variations were uncovered. Additionally, two candidate genes under the major locus were selected for further investigations. These findings will promote the research on the regulation of lignan biosynthesis in sesame, which would be useful for breeders to increase the amount of sesamin and sesamolin in sesame seeds.

## 2. Results

### 2.1. Seed Sesamin and Sesamolin Content Variation in the RILs Population

**Figure 1:**
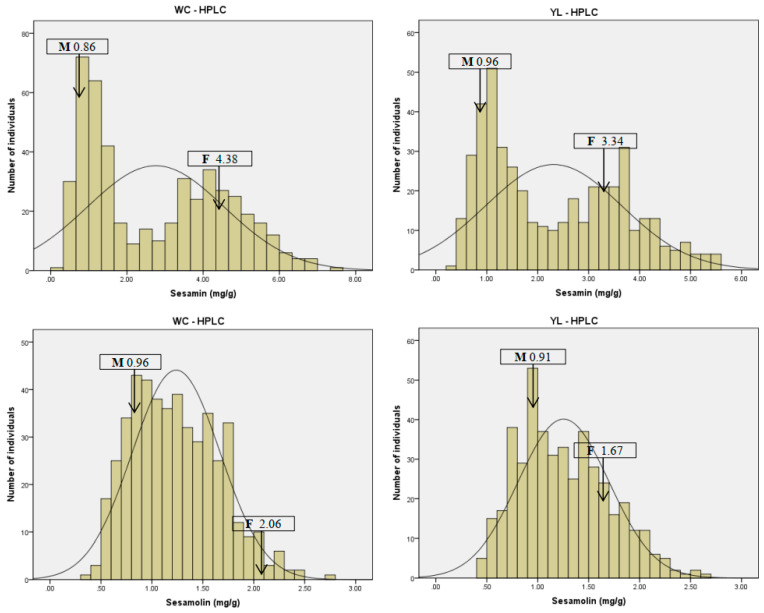
Frequency distribution of sesamin and sesamolin in Zhongzhi No. 13 × ZZM2748 RIL population of two environments. The abscissa shows the sesamin or sesamolin content, and the ordinate indicates the frequency of distribution. WC, Wuchang; YL, Yangluo; mean of two parents is indicated at the top of each histogram, with F and M representing Zhongzhi No. 13 (female parent) and ZZM2748 (male parent), respectively.

In total, seeds of 477 and 449 lines (394 commons) from Wuchang and Yangluo, respectively, were tested. The other lines were discarded from the sesamin and sesamolin contents analysis due to disease or fewer seeds. The results showed a variation of the seed sesamin and sesamolin contents among the population in the two environments. The two parents exhibited obvious differences in the two lignan contents. The averages of sesamin and sesamolin contents in Zhongzhi No. 13 seeds were 3.86 mg/g and 1.86 mg/g, respectively, and those of ZZM2748 were 0.91 mg/g and 0.94 mg/g, respectively (Table 1). Among the RIL population, the sesamin content ranged from 0.33 mg/g to 7.52 mg/g with an average of 2.76 mg/g in Wuchang, and from 0.36 mg/g to 5.58 mg/g with an average of 2.31 mg/g in Yangluo. Meanwhile, the amount of sesamolin in Wuchang and Yangluo ranged from 0.40 mg/g to 2.70 mg/g and from 0.38 mg/g to 2.70 mg/g, respectively (Table 1). The average contents of the sesamin and sesamolin in the RIL population were both between the ranges of the two parents. The datasets of sesamolin variation showed nearly normal distributions, while that of sesamin exhibited a bimodal distribution (Figure 1). The sesamin content had the highest coefficient of variation (CV) of 61.81% across the two field trials. Moreover, the sesamin and sesamolin contents showed a significant positive correlation under the two environments. As presented in Table 2, the correlation coefficient (r) between the two lignans varied from 0.64 to 0.83 (*p* 0.01).

### 2.2. The QTL for Sesamin and Sesamolin Content Variations

**Figure 2:**
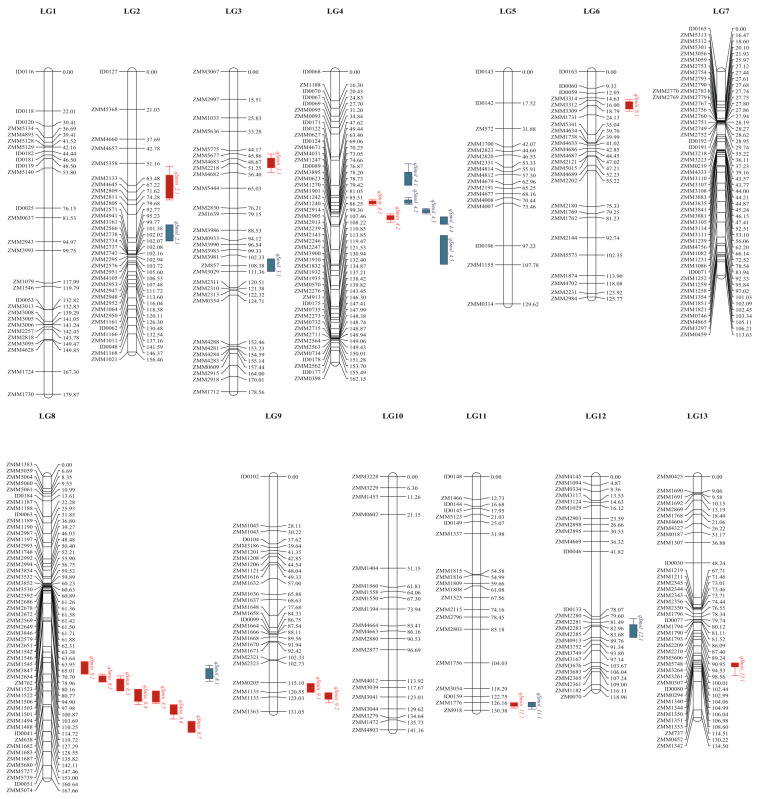
QTLs for sesamin and sesamolin positions on linkage groups of Zhongzhi No.13 × ZZM2748. Note: the red and blue bars stand for QTLs associated with sesamin and sesamolin, respectively.

The phenotype data of the 548 RILs from the two environments were used to map sesamin and sesamolin QTLs on a previously constructed genetic map by Wang et al. [29]. The genetic map constituted of 13 linkage groups comprising 424 SSR markers with a total length of 1869.78 cM. The average distance between two consecutive markers on the map was 5.1 cM. We performed the QTL mapping with two software Windows QTL Cartographer 2.5 and QTL ICIMapping v4.1 (1000 permutations, *p* = 0.05), using the composite interval mapping (CIM) method. In total, we detected 26 QTLs associated with the two lignan contents (Table 3). In particular, 16 QTLs for sesamin and 10 for sesamolin with phenotypic variation explanation (PVE) contributions of 1.15% to 67.69% and 1.87% to 46.05%, respectively. These QTLs were distributed on LG2, LG3, LG4, LG6, LG8, LG9, LG11, and LG13. The linkage groups LG4 and LG8 were outstanding, with seven and eight QTLs, respectively, accounting for half of the total loci (Figure 2). Among these QTLs, *qSmin_3.1*, *qSmin_9.2*, *qSmin_11.1* and *qSmol_11.1* were detected in the two environments. The loci *qSmin_11.1* and *qSmol_11.1* on LG11 explained 67.69% of sesamin and 46.05% of sesamolin contents variation. Notably, *qSmin11-1* and *qSmol11-1* were located in the same region as a pleiotropic locus between SSR markers ZMM1776 and ZM918 (Table 3). In order to compare the detected QTLs with the previously identified sesamin QTLs by Wu et al. [23], we mapped the RAD tags (markers) of the previous QTLs into the reference genome of Zhongzhi No. 13 [28]. The result indicated that *qSmin_4.2* might coincide with *Qsc-8* as both are located in the same interval of 3.1~4.7 Mb on Chr 4.

### 2.3. Digenic Epistatic Interactions Analysis of the Predicted Loci

**Figure 3:**
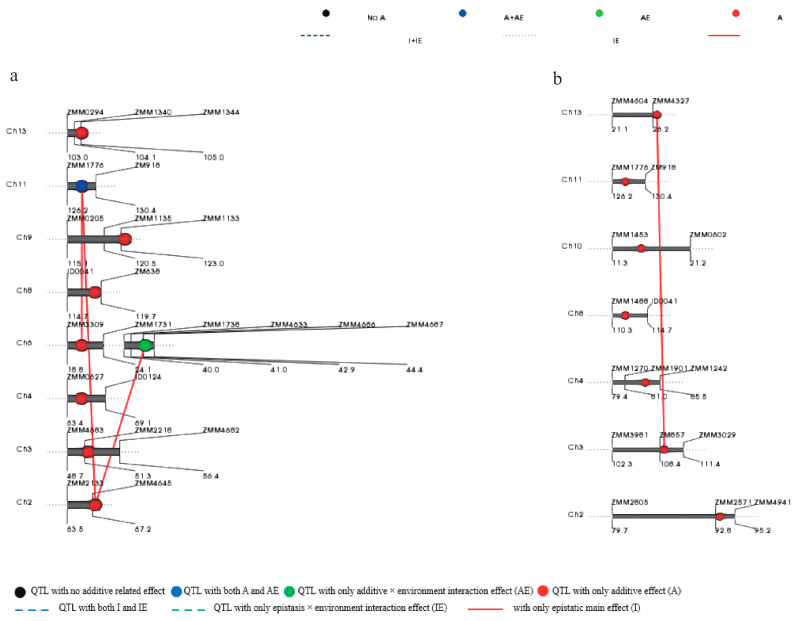
Network of epistatic QTLs for sesamin (**a**) and sesamolin (**b**) on LG2, LG3, LG6, LG11 and LG13 in RIL population detected by QTLNetwork 2.1.

To perform this analysis, we used the mixed linear model in the QTLNetwork program ver. 2.0. We identified four pairs of digenic epistatic interactions with additive × additive (AA) and AA × environment (AAE) interaction effects (Table 4). The epistatic interactions involved six loci distributed on linkage groups 2, 3, 6, 11, and 13 (Figure 3). Three pairs of loci with digenic epistatic interactions were detected for sesamin. They involved four loci that were distributed on LGs 2, 6, and 11. The AA and AAE interaction effects varied from 0.18% to 0.27% and 0.04% to 0.28%, respectively. A significant interaction effect was found between QTL *qSmin_2.1* and *qSmin_11.1* with an AA interaction effect of 0.27% and AAE of less than 0.04%. Moreover, these QTLs were implicated in the remaining two epistasis interactions for sesamin individually. Only one digenic epistatic interaction was identified for sesamolin and was found to involve two loci on LGs 3 and 13. The AA interactions contribution was 0.25%, and the AAE interactions contribution was 0.30%. 

### 2.4. Candidate Genes under the Major QTL Region

The QTL *qSmin_11.1* located near the edge of chromosome 11 between 126.2 and 129.2 cM showed pleiotropic effects for both sesamin and sesamolin contents. We screened all gene models in this region based on their functional annotation and selected 60 of them that may contain the candidate causative genes of sesamin and sesamolin. 

**Figure 4:**
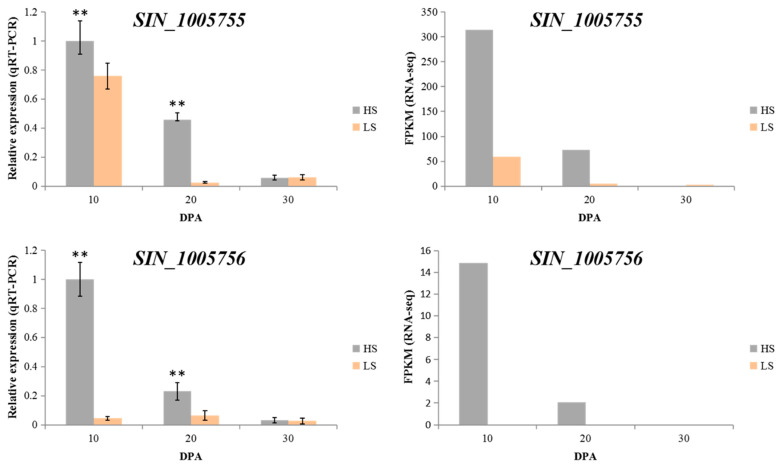
Expression profiles and qRT-PCR validation of the candidate regulatory genes. Note: HS, Zhongzhi No. 13; LS, ZZM2748. ** indicates statistically differences at *p* 0.01.

We performed the transcriptome analysis of developing seed at 10, 20, and 30 DPA (days post-anthesis) and compared the expression profiles of the 60 preselected genes. Among these genes, only *SIN_1005755* and *SIN_1005756* showed significant expression differences in the two parents at 10, 20, and 30 DPA with the highest expression in Zhongzhi No.13. In particular, *SIN_1005756* was not expressed in ZZM2748 (Figure 4; Supplementary Figure S1). To check the reliability of the RNA-seq data, we performed the qRT-PCR of the two candidate genes. The results were consistent with the RNA-seq with similar trends, supporting the RNA-seq analysis (Figure 4). We then selected *SIN_1005755* and *SIN_1005756* as possible candidate genes associated with sesamin and sesamolin variation in sesame developing seed for future studies. *SIN_1005755* was annotated as the NAC domain-containing protein in the Swissprot database. *SIN_1005756* was a novel gene without any annotation (Table S1). 

Moreover, the expression profiles of the five genes that may be involved in sesame lignan biosynthesis were also checked [26]. The five genes included a dirigent gene (*SIN_1015471*), a piperitol/sesamin synthase (PSS) gene (*SIN_1025734*) and three homologous genes of PSS—*SIN_1025729*, *SIN_1025730* and *SIN_1003948*. *SIN_1003948* was expressed lower in the two parents both at 10, 20, and 30 DPA. No significant difference was observed in the expression profile of *SIN_1015471* and *SIN_1025734* in the two parents. *SIN_1025729* was expressed differently at 30 DPA in the two parents. *SIN_1025730* showed high expression in ZZM2748 at the three stages in comparison with Zhongzhi No. 13.

## 3. Discussion

Sesame breeding programs have been focused mostly on seed yield, disease resistance, and high oil content. Recently, the objective in sesame breeding has been changed due to the discovery of huge positive effects of sesame lignans on human and animal health. Currently, breed environment stable sesame varieties containing high oil and lignans are the focus of sesame breeders. Previous studies demonstrated that genomic assisted-breeding techniques could be useful for creating high-quality varieties in sesame [37]. However, the genetic basis of lignan content in sesame is still not well-understood. Therefore, acknowledging the genetic control of sesamin and sesamolin biosynthesis in sesame is of the utmost interest. Quantitative trait locus (QTL) mapping is a common practice in crop plants due to progress made in the statistical genomics and molecular markers area [32]. It was useful in detecting loci linked to complex quantitative traits in sesame and other crops [12,32]. The present study investigated the variation of 548 RIL seed sesamin and sesamolin contents in two environments and revealed 26 loci associated with the two lignans in sesame. 

Several studies conducted on the variability of lignans content proved that sesamin and sesamolin are the major lignans that constitute sesame seed [19,38,39]. Generally, sesamin accounts for 0.20 to 8.00 mg/g of the dry weight of sesame seeds. To our knowledge, this is the first report on the variability of sesamin and sesamolin contents in a RIL population grown in two distinct locations using the HPLC method. In the two environments, the 548 RILs seed sesamin and sesamolin contents varied from 0.33~7.52 mg/g and 0.36~2.70 mg/g, respectively, and those for the two parents ranged from 0.86~4.38 mg/g and 0.91~2.06 mg/g, respectively. The results suggested the possibility to obtain higher lignans content sesame lines from the cross of proper parents, although the efficiency of traditional crossbreeding in sesame is low. The results agree with the reports on the variability of sesamin and sesamolin content observed in various germplasms. Wu et al. [23] used near-infrared reflectance (NIR) spectroscopy to evaluate sesame seed sesamin content in 224 RILs from three locations and observed a variation from 1.70~5.10 mg/g. Wang et al. [16] reported that seed sesamin and sesamolin mainly ranged from 0.88~11.05 mg/g and 0.93~6.96 mg/g, respectively, in a core collection conserved in China. In some Indian and Thai germplasms, seed sesamin ranged from 0.08~6.45 mg/g and 1.63~7.23 mg/g, respectively, and sesamolin from 0.28~3.76 mg/g and 0.48~2.25 mg/g, respectively [19,40,41]. The phenotype data from the two locations showed a significant positive correlation between sesamin and sesamolin content. The CV of sesamin in Wuchang and Yangluo was 65.12% and 58.03%, respectively, and that of sesamolin was 34.85% and 35.53%, respectively. These results are consistent with previous reports supporting that sesamin and sesamolin contents in seeds are influenced by environmental conditions [18,19]. 

Despite the importance of seed sesamin and sesamolin contents in sesame genotypes selection, few genomic markers were detected to be associated with these lignans variation. Using multi-locus mapping, Lei et al. [36] detected eight significant SNPs associated with sesamin (M15E10-5, M7E18-2, SSI182-3, and SSR023-1) and sesamolin (E5M6-3, M8E10-1, SSI182-3, and SSI281-4). The PVE of the sesamin and sesamolin SNPs ranged from 3.33% to 6.36% and 3.30% to 5.21%, respectively. Wu et al. [23] combined mixed composite interval mapping (MCIM) and multiple interval mapping methods in 224 RILs grown in three environments and detected five QTLs for sesamin. These QTLs were distributed on LGs 5, 6, 8, 11, and 16 and explained 0.41% to 14.55% of PVE. Here, using CIM in two software (Windows QTL Cartographer 2.5 and QTL IciMapping v4.1) and phenotype data from 548 RILs cultivated in two locations, we identified 16 and 10 QTLs linked to sesamin and sesamolin, respectively. The PVE of sesamin and sesamolin QTLs varied from 1.15% to 67.69% and 1.87% to 46.05%, respectively. The 26 QTLs were distributed on all the LGs except LGs 1, 5, and 7. The comparison of the location of the detected QTLs with the previously identified sesamin QTLs by Wu et al. [23] indicated that *qSmin_4.2* might be identical to *Qsc-8*. These results indicate the need for functional characterization of the identified QTLs to confirm their potential effect on sesamin and sesamolin contents variation in sesame.

Major QTLs with high heritability detected simultaneously in multiple environments are considered highly stable and reliable. We detected one major pleiotropic QTLs (*qSmin_11.1/qSmol_11.1*) related to sesamin and sesamolin. The QTLs were located between the same SSR markers (ZMM1776 and ZM918) on LG11 and explained 67.69% and 46.05% of the PVE, respectively. Of the remaining 24 minor QTLs, seven and eight QTLs were located closely on LG4 and LG8, respectively, indicating they could originate from one true QTL on the respective LGs. These findings support the close genetic correlation between sesamin and sesamolin and suggest epistatic effects in genetic control of lignan biosynthesis in sesame. Therefore, we performed the digenic epistatic interactions analysis and identified three and one pairs of epistatic interactions with AA and AAE effects for sesamin and sesamolin, respectively. The major QTL *qSmin_11.1* was involved in two pairs of epistatic interactions with AA interaction effects of 0.18% to 0.27% and AAE interaction effects of 0.04% to 0.22%. These results were in accordance with previous studies stating that sesamin and sesamolin contents are polygenic traits controlled by additive and dominance effects [13,14,15]. Moreover, they confirmed that the yield of these lignans could be affected by growth conditions. Two new loci that were not identified by the QTL mapping were revealed by the QTLNetwork. Such loci might be minor QTLs that are generally difficult to detect by mapping [32]. Although minor QTLs are unstable, they can influence lignan biosynthesis through AA and AAE interactions. It also suggested that cloning of all the identified QTL regions can help to dissect the genetic basis of sesamin and sesamolin contents in sesame.

Previous studies reported that the biosynthesis of sesame lignans might involve complex biochemical mechanisms [42,43,44]. The expression profiles of pineresinol and piperitol/sesamin synthase genes (*SIN_1015471*, *SIN_1025734*, *SIN_1025729*, *SIN_1025730*, *SIN_1003948*) observed in this study confirmed that these genes were not involved in the regulation of sesamin and sesamolin content variations. Coupling transcriptome analysis of the parent’s developing seed at 10, 20, and 30 DPA with gene function-annotation, we screened the major QTL region and selected two candidate genes (*SIN_1005755* and *SIN_1005756*) that might control sesamin and sesamolin biosynthesis. *SIN_1005755* encoded an NAC domain-containing protein, while *SIN_1005756* was a novel gene without any annotation. NAC proteins are transcription regulatory genes and are involved in various gene interaction regulatory networks [45]. Studies in rice, pepper, populus, cotton, and Arabidopsis indicated that NAC domain proteins influence lignin biosynthesis and composition, promote senescence by inducing chlorophyll degradation, regulate abiotic stress responses and secondary cell wall biosynthesis, and can be targeted to obtain nutrient remobilization in crop plants [45,46,47,48,49]. This suggests that *SIN_1005755* might be involved in sesamin biosynthesis regulation by controlling lignification in the sesame seed coat. Thus, functional studies using advanced genome editing tools are needed to dissect the roles of these genes, especially *SIN_1005755,* in the lignans pathway and during sesame seed development. Our findings constitute a foundation for further investigations that will lead to genomic-assisted breeding of high-quality sesame varieties.

## 4. Materials and Methods

### 4.1. Plant Materials

A population of 548 recombinant inbred lines (RILs, F8) derived from a cross between ZZM2748 (P1, male parent) and Zhongzhi No. 13 (P2, female parent) was used for sesamin and sesamolin contents related QTL mapping in this study. Zhongzhi No. 13 had been de novo sequenced [28]. All the plant materials were given by the National Medium-term Sesame GenBank of China (Wuhan, China).

### 4.2. Experimental Design and Sampling

The RILs and the two parents were cultivated in two experimental field stations of the Oil Crops Research Institute of the Chinese Academy of Agricultural Sciences (OCRI-CAAS) located in Wuchang and Yangluo (Hubei province, China). The Wuhan field trial was carried out in 2013 and that of Yangluo in 2014. In each location, all the genotypes were grown in a complete randomized block design with three replications. Standard agronomic practices were used in field management. The seeds were harvested in the two trials when they reached maturity. The three replicate seeds from each location were mixed equally and well-preserved at the seed storage room of OCRI up to the high-performance liquid chromatography (HPLC) analysis of sesamin and sesamolin contents.

### 4.3. Sesamin and Sesamolin Extraction from Seeds

The sample sesamin and sesamolin were extracted following the method of Rangkadilok et al. [41], modified by Wang et al. [16]. In brief, 0.6–0.7 g of seed sample was ground to a fine powder with a mortar containing liquid nitrogen. The sample’s flour was accurately weighed (200 mg) and dissolved in 5.0 mL of 80% ethanol after its temperature returned to normal. Then, the samples were vortex-mixed for two hours and centrifuged for 5 min at 5000 rpm. The supernatant was transferred into a 15 mL volumetric flask, and the residue was re-extracted with 5.0 mL of 80% ethanol. Finally, the two extracted solutions were mixed and filtered with a 0.22 µm Nylon membrane prior to HPLC analysis.

### 4.4. HPLC Analysis of Sesamin and Sesamolin

Using the standard external method, the extractions were analyzed by Agilent 1260 Infinity II (HPLC, Agilent Technologies, Waldbronn, Germany) with a thermostatically controlled column oven, a binary pump, and a diode-array detector as per Wang et al. [16]. The reversed-phase column consisted of Agilent ZORBAX SB-C18 (250 mm × 4.6 mm, 5 μm). The mobile phase was a mixture of methanol-deionized water (80/20, v/v) at a flow rate of 1 mL/min (injection volume 10 µL). The absorption was monitored at 290 nm. Each sample was checked twice, and the average was counted as the final contents of sesamin and sesamolin. When the discrepancy between the two repeats was 10% higher than the average, the sample’s analysis was repeated.

### 4.5. Phenotypic Data Analysis

The descriptive statistics, correlation analysis, and frequency distribution were performed using IBM SPSS Statistics 20 for Windows (SPSS Inc, Chicago, IL, USA). Additionally, phenotypic data for sesamin and sesamolin were recorded using the Microsoft Office Excel 2010 software (https://www.microsoft.com).

### 4.6. QTL Analysis

The genetic map constructed previously from the same RIL population was associated with the sesamin and sesamolin content variations [29]. It covered a total map length of 1869.78 cM of the sesame genome with 424 SSR markers clustered in 13 linkage groups. To ensure the reliability of the QTL detection, the QTL analysis was performed with Windows QTL Cartographer version 2.5 (Microsoft, Inc., Redmond, WA, USA) and QTL IciMapping version 4.1 (1000 permutations, *p* = 0.05) using the composite interval mapping (CIM) algorithm. In Windows QTL Cartographer, the experiment-wide threshold was determined by 1000 permutations at a significance level of 0.05 with a genome walk speed of 1 cM. Marker IDs, LOD scores, and genetic positions were collated on a per-trait basis and imported to MapChart version 2.32 for QTL visualization [50]. The detected QTLs were named as suggested by McCouch et al. [51]. The designation started with a “q” (lowercase), then the trait name in capital letters. Since the two traits have the same initial “S”, “min” and “mol” were added after the abbreviation “S” to differentiate sesamin and sesamolin, respectively. Finally, the chromosome and the serial number numbers were added.

The digenic epistatic interactions between the Loci, with their AA and AAE effects, were analyzed by QTLNetwork software version 2.1 [52]. The testing window, walking speed, and filtration window were set at 10, 1, and 10 cM, respectively. The permutation test was 1000 times, and all significance level configurations were 0.05.

### 4.7. RNA Isolation and RNA-seq Analysis

To examine the gene expression difference between the two parents, developing seeds of P1 and P2 at 10, 20, and 30 DPA were sampled in triplicate from the Wuchang field for RNA-seq. The total RNA was extracted from each sample using Trizol reagent. The RNA quality was checked on a Bioanalyzer 2100 (Aligent, Santa Clara, CA, USA); the RNA integrity number (RIN) values were ≥7. RNA-Seq libraries were prepared and sequenced on an Illumina HiSeq2500 platform. The gene expression levels were normalized to the number of fragments per kilobase of transcript per million mapped reads (FPKM) using the HTSeq 6.0 software [53].

### 4.8. qRT-PCR Validation

Real-time quantitative PCR was performed using SYBR^®^ Select Master Mix (2X) (Vazyme Biotec, Nanjing, China) on a Light Cycler 480 II (Roche, Basel, Switzerland). Specific primers for the selected genes were designed using Primer Premier 5.0 (Table S1). Histone H3.3 gene (*SIN_1004293*) was used as the internal control to normalize transcript levels. The PCR reaction mixtures were as follows: 10 μL mix (Vazyme Biotec, Nanjing, China), 5 µL cDNA, 0.5 µL of each primer, and 4 µL ddH_2_O. The PCR program was conducted according to the manufacturer’s protocol. Pre-incubation, one cycle: 95 ℃ for 30 s; amplification, 40 cycles: 95 ℃ for 10 s, 60 ℃ for 30 s; melting curve, one cycle: 95 ℃ for 15 s, 60 ℃ for 60 s, 95 ℃ for 15 s; cooling, one cycle: 40 ℃ for 30 s. The real-time assay for each gene was performed with three independent biological replicates under identical conditions. Each gene expression level was calculated from cycle threshold values using the 2^−ΔΔCt^ method [54].

## 5. Conclusions

In summary, we investigated the variability of sesamin and sesamolin in 548 RILs cultivated in two environments. It showed broad variation with some lines over the high parent in sesamin or sesamolin content, indicating that these traits could be easily improved. A total of 26 QTLs associated with sesamin and sesamolin contents variations were detected, including a locus (*qSmin_11.1*/*qSmol_11.1*) of large effect. This locus and its flanking SSR markers might be suitable and tested for marker-assisted selection. The QTLNetwork analysis confirmed that sesamin and sesamolin contents are regulated by epistatic interactions with AA and AAE effects. Finally, two candidate genes (*SIN_1005755*, *SIN_1005756*) that might be associated with these lignans variation were selected and will be targeted for functional studies to understand the molecular mechanisms involved in lignan biosynthesis in sesame and for genomics-assisted breeding of varieties containing high sesamin and sesamolin.

## Figures and Tables

**Table 1:** Phenotypes of sesamin and sesamolin in RIL families and parental lines.

Traits	Location	Parent		RIL Population					
		Zhongzhi No. 13	ZZM2748	Mean + sd	Maximum	Minimum	Kurtosis	Skew	CV (%)
Sesamin (mg/g)	Wuchang	4.38	0.86	2.76 ± 1.8	7.52	0.33	−1.15	0.39	65.12
	Yangluo	3.34	0.96	2.31 ± 1.34	5.58	0.36	−0.99	0.46	58.03
	Mean	3.86	0.91	2.54 ± 1.57	6.55	0.35	−1.07	0.42	61.81
Sesamolin (mg/g)	Wuchang	2.06	0.96	1.24 ± 0.43	2.70	0.36	−0.31	0.43	34.85
	Yangluo	1.67	0.91	1.25 ± 0.45	2.70	0.40	−0.33	0.49	35.53
	Mean	1.86	0.94	1.25 ± 0.44	2.70	0.38	−0.31	0.47	35.19

Note: sd—standard deviation; CV—coefficient of variation.

**Table 2:** Pairwise correlation coefficients between sesamin and sesamolin across two environments.

Trait	Location	Sesamin		Sesamolin	
		Wuchang	Yangluo	Wuchang	Yangluo
Sesamin	Wuchang	1			
	Yangluo	0.834 **	1		
Sesamolin	Wuchang	0.765 **	0.640 **	1	
	Yangluo	0.668 **	0.789 **	0.728 **	1

** indicates significant correlation at the 0.01 probability level.

**Table 3:** Additive effect QTLs for sesamin and sesamolin based on phenotyping under two environments Wuchang, Yangluo, and detected using Windows QTL Cartographer version 2.5 and QTL IciMapping 4.1.

Trait	QTL	Chr	Position (cM)	Left Marker	Right Marker	LOD	Add	R^2^ (%)	LOD_L (cM)	LOD_R (cM)	WinQTLcart		ICIMapping	
											WC	YL	WC	YL
sesamin	*qSmin_2.1*	2	66.51–66	ZMM2133	ZMM4645	3.54	−0.20	1.20	52.8	71.6	√		√	
	*qSmin_3.1*	3	50.71	ZMM4683	ZMM2218	4.22	−0.19	1.42	46.0	56.2	√	√		
	*qSmin_4.1*	4	73–73.11	ZMM4671	ZMM4031	4.34	0.17	1.45	71.5	74.7		√		√
	*qSmin_4.2*	4	81.11	ZMM1901	ZMM1242	3.77	0.15	1.29	79.4	84.0		√		
	*qSmin_6.1*	6	18.01–20	ZMM3312	ZMM1731	4.56	0.23	1.79	15.6	22.1	√		√	
	*qSmin_8.1*	8	106.71	ZMM1494	ZMM1488	3.37	0.20	1.25	103.2	110.3	√			
	*qSmin_8.2*	8	114.31	ZMM1488	ID0041	4.74	0.23	1.64	110.3	114.7	√			
	*qSmin_8.3*	8	116.71–118	ID0041	ZM638	6.45	0.25	2.53	111.3	119.7		√	√	
	*qSmin_8.4*	8	120.71	ZM638	ZMM1682	5.20	0.25	1.82	118.7	127.3	√			
	*qSmin_8.5*	8	122.71	ZM638	ZMM1682	5.98	0.22	2.51	119.7	126.7		√		
	*qSmin_8.6*	8	128.61	ZMM1683	ZMM1687	4.65	0.22	1.53	127.4	135.2	√			
	*qSmin_8.7*	8	139	ZMM1687	ZMM5680	5.52	0.21	1.23	136.5	142.5				√
	*qSmin_9.1*	9	119	ZMM0205	ZMM1135	6.20	−0.22	1.29	115.5	120.5				√
	*qSmin_9.2*	9	122.61–123.01	ZMM1135	ZMM1133	8.78	−0.29	2.67	120.6	126.2	√	√	√	
	*qSmin_11.1*	11	127–127.21	ZMM1776	ZM918	108.38	1.27	67.69	126.2	129.2	√	√	√	√
	*qSmin_13.1*	13	105.01	ZMM1344	ZMM1350	3.88	0.20	1.15	104.0	111.1	√		√	
Sesamolin	*qSmol_2.1*	2	91	ZMM2805	ZMM2571	3.19	0.06	2.02	85.5	94.5				√
	*qSmol_3.1*	3	108	ZMM3981	ZM857	4.57	0.07	2.31	104.5	111.5			√	
	*qSmol_4.1*	4	57.51	ID0122	ZMM0627	3.28	0.07	2.16	51.5	63.4		√		
	*qSmol_4.2*	4	72.31–73	ZMM4671	ZMM4031	6.18	0.08	4.04	70.4	74.1		√		√
	*qSmol_4.3*	4	77.91	ID0089	ZMM3895	6.12	0.08	3.09	76.4	79.4		√		
	*qSmol_4.4*	4	83.11–85	ZMM1901	ZMM1242	5.26	0.07	3.82	81.1	85.2		√	√	
	*qSmol_4.5*	4	102	ZMM2914	ZMM2905	3.57	0.07	2.02	91.5	107.5				√
	*qSmol_8.1*	8	109.71–111	ZMM1494	ID0041	4.26	0.07	2.53	105.4	114.3	√		√	
	*qSmol_11.1*	11	127–127.21	ZMM1776	ZM918	63.45	0.30	46.05	126.0	129.8	√	√	√	√
	*qSmol_12.1*	12	87.71	ZMM2285	ZMM0913	3.52	−0.06	1.87	79.4	89.8		√		

Chr—chromosome number; LOD—logarithm of odds for each QTL; Add—additive effect; R^2^—contribution rate.

**Table 4:** Digenic epistatic interactions detected for sesamin and sesamolin in the RIL population in two environments.

Trait	QTL_i	Interval_i	Position_i	Range_i	QTL_j	Interval_j	Position_j	Range_j	AA	AAE1	AAE2	H^2^ (AA), %	H^2^ (AAE), %
sesamin	2–6	ZMM2133–ZMM4645	66.5	63.5–67.2	6–11	ZMM4633–ZMM4686	42	41.0–42.9	−0.0773	−0.0504	0.0506	0.22	0.28
	2–6	ZMM2133–ZMM4645	66.5	63.5–67.2	11–19	ZMM1776–ZM918	127.2	126.2–128.2	−0.0848	−0.0001	0.0001	0.27	0.04
	6–6	ZMM3312–ZMM1731	19.8	18.8–21.8	11–19	ZMM1776–ZM918	127.2	126.2–128.2	0.0819	0.0446	−0.0445	0.18	0.22
sesamolin	3–17	ZMM3981–ZM857	108.3	105.3–108.4	13–7	ZMM4604–ZMM4327	26.1	22.1–26.2	0.0244	−0.0107	0.0107	0.25	0.03

Note: QTL_i and QTL_j—The two QTLs involved in the epistatic interaction. interval_i—the flanking markers of QTL_i. interval_j—the flanking markers of QTL_j. AA—additive × additive (AA) interaction effect. AAE1, AAE2—AA × environment (AAE) interaction effects in Wuchang (E1) and Yangluo (E2), respectively. H^2^ (AA)—the heritability of AA interaction effects. H^2^ (AAE)—the heritability of AAE interaction effects.

## Data Availability

The datasets generated during and/or analyzed during the current study are available from the corresponding author on reasonable request.

## Supplementary Materials

The following are available online at https://www.mdpi.com/article/10.3390/plants10071343/s1: Figure S1: the expression profile of 20 genes, including the candidate regulatory genes in Zhongzhi No. 13 and ZZM2748, at three stages of seed development; Table S1: primer sequence used for qRT-PCR and functional annotation of the candidate regulatory genes.

## Abbreviations

HPLC, high-performance liquid chromatography; RIL, recombinant inbred line; CIM, composite interval mapping; QTL, quantitative trait locus; LOD, logarithm of odds; LG, linkage group; SSR, simple sequence repeat; NIR, near-infrared reflectance; AA, additive × additive; AAE, AA × environment; DPA, days post-anthesis; CV, coefficient of variation; cM, centimorgan; PCR, polymerase chain reaction.

## References

[1] Wan Y., Li H., Fu G., Chen X., Chen F., Xie M. (2015). The relationship of antioxidant components and antioxidant activity of sesame seed oil. J. Sci. Food Agric..

[2] Satake H., Koyama T., Bahabadi S.E., Matsumoto E., Ono E., Murata J. (2015). Essences in metabolic engineering of lignan biosynthesis. Metabolites.

[3] Pathak N., Rai A.K., Kumari R., Bha K.V. (2014). Value addition in sesame: A perspective on bioactive components for enhancing utility and profitability. Pharmacogn. Rev..

[4] Kim A.Y., Yun C.I., Lee J.G., Kim Y.J. (2020). Determination and Daily Intake Estimation of Lignans in Sesame Seeds and Sesame Oil Products in Korea. Foods.

[5] Davin L.B., Lewis N.G. (2005). Dirigent phenoxy radical coupling: Advances and challenges. Curr. Opin. Biotechnol..

[6] Budowski P. (1964). Recent Research on Sesamin, Sesamolin, and Related Compounds. J. Am. Oil Chem. Soc..

[7] Majdalawieh A.F., Dalibalta S., Yousef S.M. (2020). Effects of sesamin on fatty acid and cholesterol metabolism, macrophage cholesterol homeostasis and serum lipid profile: A comprehensive review. Eur. J. Pharmacol..

[8] Majdalawieh A.F., Massri M., Nasrallah G.K. (2017). A comprehensive review on the anti-cancer properties and mechanisms of action of sesamin, a lignan in sesame seeds (*Sesamum indicum*). Eur. J. Pharmacol..

[9] Kim Y.H., Kim E.Y., Rodriguez I., Nam Y.H., Jeong S.Y., Hong B.N., Choung S.Y., Kang T.H. (2020). *Sesamum indicum* L. Oil and Sesamin Induce Auditory-Protective Effects through Changes in Hearing Loss-Related Gene Expression. J. Med. Food.

[10] Abe-Kanoh N., Kunimoto Y., Takemoto D., Ono Y., Shibata H., Ohnishi K., Kawai Y. (2019). Sesamin Catechol Glucuronides Exert Anti-inflammatory Effects by Suppressing Interferon β and Inducible Nitric Oxide Synthase Expression through Deconjugation in Macrophage-like J774.1 Cells. J. Agric. Food Chem..

[11] Kim K.-S., Lee J.-R., Lee J.-S. (2006). Determination of sesamin and sesamolin in sesame (*Sesamum indicum* L.) seeds using UV spectrophotometer and HPLC. Korean J. Crop Sci..

[12] Dossa K., Diouf D., Wang L., Wei X., Zhang Y., Niang M., Fonceka D., Yu J., Mmadi M.A., Yehouessi L.W. (2017). The Emerging Oilseed Crop *Sesamum indicum* Enters the “Omics” Era. Front. Plant Sci..

[13] Umezawa T. (2003). Diversity in lignan biosynthesis. Phytochem. Rev..

[14] Ke T., Dong C., Mao H., Zhao Y., Chen H., Liu H., Dong X., Tong C., Liu S. (2011). Analysis of expression sequence tags from a full-length-enriched cDNA library of developing sesame seeds (Sesamum indicum). BMC Plant Biol..

[15] Usman S.M., Viswanathan P.L., Manonmani S., Uma D. (2020). Genetic studies on sesamin and sesamolin content and other yield attributing characters in sesame (*Sesamum indicum* L.). Electron. J. Plant Breed..

[16] Wang L., Zhang Y., Li P., Wang X., Zhang W., Wei W., Zhang X. (2012). HPLC Analysis of Seed Sesamin and Sesamolin Variation in a Sesame Germplasm Collection in China. J. Am. Oil Chem. Soc..

[17] Ajit G., Uma D., Manonmani S., Vinothkumar B., Rajesh S. (2019). Diversity Analysis of Sesame Lignans in 40 Sesame Collections in Tamil Nadu, India. Int. J. Curr. Microbiol. Appl. Sci..

[18] Kim S.-U., Oh K.-W., Lee M.-H., Lee B.-K., Pae S.-B., Hwang C.-D., Kim M.-S., Baek I.-Y., Lee J.-D. (2014). Variation of Lignan Content for Sesame Seed Across Origin and Growing Environments. Korean J. Crop Sci..

[19] Dar A.A., Kancharla P.K., Chandra K., Sodhi Y.S., Arumugam N. (2019). Assessment of variability in lignan and fatty acid content in the germplasm of *Sesamum indicum* L.. J. Food Sci. Technol..

[20] Li C., Miao H., Wei L., Zhang T., Han X., Zhang H. (2014). Association mapping of seed oil and protein content in *Sesamum indicum* L. using SSR markers. PLoS ONE.

[21] Kancharla P.K., Arumugam N. (2020). Variation of Oil, Sesamin, and Sesamolin Content in the Germplasm of the Ancient Oilseed Crop *Sesamum indicum* L.. J. Am. Oil Chem. Soc..

[22] Wei X., Liu K., Zhang Y., Feng Q., Wang L., Zhao Y., Li D., Zhao Q., Zhu X., Zhu X. (2015). Genetic discovery for oil production and quality in sesame. Nat. Commun..

[23] Wu K., Wu W.-X., Yang M.-M., Liu H.-Y., Hao G.-C., Zhao Y.-Z. (2017). QTL Mapping for Oil, Protein and Sesamin Contents in Seeds of White Sesame. Acta Agron. Sin..

[24] Ghotbzadeh Kermani S., Saeidi G., Sabzalian M.R., Gianinetti A. (2019). Drought stress influenced sesamin and sesamolin content and polyphenolic components in sesame (*Sesamum indicum* L.) populations with contrasting seed coat colors. Food Chem..

[25] Sato F., Matsui K. (2012). Engineering the biosynthesis of low molecular weight metabolites for quality traits (essential nutrients, health-promoting phytochemicals, volatiles, and aroma compounds). Plant Biotechnology and Agriculture.

[26] Ono E., Nakai M., Fukui Y., Tomimori N., Fukuchi-Mizutani M., Saito M., Satake H., Tanaka T., Katsuta M., Umezawa T. (2006). Formation of two methylenedioxy bridges by a *Sesamum* CYP81Q protein yielding a furofuran lignan, (+)-sesamin. Proc. Natl. Acad. Sci. USA.

[27] Murata J., Ono E., Yoroizuka S., Toyonaga H., Shiraishi A., Mori S., Tera M., Azuma T., Nagano A.J., Nakayasu M. (2017). Oxidative rearrangement of (+)-sesamin by CYP92B14 co-generates twin dietary lignans in sesame. Nat. Commun..

[28] Wang L., Yu S., Tong C., Zhao Y., Liu Y., Song C., Zhang Y., Zhang X., Wang Y., Hua W. (2014). Genome sequencing of the high oil crop sesame provides insight into oil biosynthesis. Genome Biol..

[29] Wang L., Zhang Y., Zhu X., Zhu X., Li D., Zhang X., Gao Y., Xiao G., Wei X., Zhang X. (2017). Development of an SSR-based genetic map in sesame and identification of quantitative trait loci associated with charcoal rot resistance. Sci. Rep..

[30] Wu K., Liu H., Yang M., Tao Y., Ma H., Wu W., Zuo Y., Zhao Y. (2014). High-density genetic map construction and QTLs analysis of grain yield-related traits in Sesame (*Sesamum indicum* L.) based on RAD-Seq techonology. BMC Plant Biol..

[31] Wang L., Zhang Y., Li D., Dossa K., Wang M.L., Zhou R., Yu J., Zhang X. (2019). Gene expression profiles that shape high and low oil content sesames. BMC Genet..

[32] Kulwal P.L. (2018). Trait Mapping Approaches through Linkage Mapping in Plants. Adv. Biochem. Eng. Biotechnol..

[33] Zhang H., Miao H., Wei L., Li C., Zhao R., Wang C. (2013). Genetic analysis and QTL mapping of seed coat color in sesame (*Sesamum indicum* L.). PLoS ONE.

[34] Zhang Y., Wang L., Li D., Gao Y., Lu H., Zhang X. (2014). Mapping of Sesame Waterlogging Tolerance QTL and Identification of Excellent Waterlogging Tolerant Germplasm. Sci. Agric. Sin..

[35] Rao P.V.R., Prasuna K., Anuradha G., Srividya A., Vemireddy L.R., Shankar V.G., Sridhar S., Jayaprada M., Reddy K.R., Reddy N.P.E. (2014). Molecular mapping of important agro-botanic traits in sesame. Electron. J. Plant Breed..

[36] Wang L., Li D., Xi X., Zhang Y., Ding X., Wang L., Wei W., Gao Y., Zhang X. (2014). Association analysis of sesamin and sesamolin in the core sesame (*Sesamum indicum* L.) germplasm. Chin. J. Oil. Crop. Sci..

[37] Tiwari S., Kumar S., Gontia I. (2011). Biotechnological approaches for sesame (*Sesamum indicum* L.) and niger (*Guizotia abyssinica* L.f. Cass.). Asia-Pac. J. Mol. Biotechnol..

[38] Dar A.A., Arumugam N. (2013). Lignans of sesame: Purification methods, biological activities and biosynthesis—A review. Bioorg. Chem..

[39] Pathak N., Rai A.K., Saha S., Walia S., Sen S.K., Bhat K.V. (2014). Quantitative dissection of antioxidative bioactive components in cultivated and wild sesame germplasm reveals potentially exploitable wide genetic variability. J. Crop Sci. Biotechnol..

[40] Muthulakshmi C., Pavithra S., Selvi S. (2017). Evaluation of sesame (*Sesamum indicum* L.) germplasm collection of Tamil Nadu for Î±-linolenic acid, sesamin and sesamol content. Afr. J. Biotechnol..

[41] Rangkadilok N., Pholphana N., Mahidol C., Wongyai W., Saengsooksree K., Nookabkaew S., Satayavivad J. (2010). Variation of sesamin, sesamolin and tocopherols in sesame (*Sesamum indicum* L.) seeds and oil products in Thailand. Food Chem..

[42] Murata J., Matsumoto E., Morimoto K., Koyama T., Satake H. (2015). Generation of Triple-Transgenic Forsythia Cell Cultures as a Platform for the Efficient, Stable, and Sustainable Production of Lignans. PLoS ONE.

[43] Pathak N., Bhaduri A., Bhat K.V., Rai A.K. (2015). Tracking sesamin synthase gene expression through seed maturity in wild and cultivated sesame species—A domestication footprint. Plant Biol..

[44] Tera M., Koyama T., Murata J., Furukawa A., Mori S., Azuma T., Watanabe T., Hori K., Okazawa A., Kabe Y. (2019). Identification of a binding protein for sesamin and characterization of its roles in plant growth. Sci. Rep..

[45] Podzimska-Sroka D., O’Shea C., Gregersen P.L., Skriver K. (2015). NAC Transcription Factors in Senescence: From Molecular Structure to Function in Crops. Plants.

[46] Guo W.L., Wang S.B., Chen R.G., Chen B.H., Du X.H., Yin Y.X., Gong Z.H., Zhang Y.Y. (2015). Characterization and expression profile of CaNAC2 pepper gene. Front. Plant Sci..

[47] Sun Q., Huang J., Guo Y., Yang M., Guo Y., Li J., Zhang J., Xu W. (2020). A cotton NAC domain transcription factor, GhFSN5, negatively regulates secondary cell wall biosynthesis and anther development in transgenic Arabidopsis. Plant Physiol. Biochem..

[48] Hu P., Zhang K., Yang C. (2019). BpNAC012 Positively Regulates Abiotic Stress Responses and Secondary Wall Biosynthesis. Plant Physiol..

[49] Yang Y., Yoo C.G., Rottmann W., Winkeler K.A., Collins C.M., Gunter L.E., Jawdy S.S., Yang X., Pu Y., Ragauskas A.J. (2019). PdWND3A, a wood-associated NAC domain-containing protein, affects lignin biosynthesis and composition in Populus. BMC Plant Biol..

[50] Voorrips R.E. (2002). MapChart: Software for the graphical presentation of linkage maps and QTLs. J. Hered..

[51] Mccouch S.R., Cho Y.G., Yano M., Paul E., Blinstrub M., Morishima H., Kinosita T., McCouch S.R., Cho Y.G., Yano M. (1997). Report on QTL nomenclature. Rice Genet. Newsl..

[52] Yang J., Hu C., Hu H., Yu R., Xia Z., Ye X., Zhu J. (2008). QTLNetwork: Mapping and visualizing genetic architecture of complex traits in experimental populations. Bioinformatics.

[53] Anders S., Huber W. (2010). Differential expression analysis for sequence count data. Genome Biol..

[54] Livak K.J., Schmittgen T.D. (2001). Analysis of relative gene expression data using real-time quantitative PCR and the 2^−ΔΔCT^ Method. Methods.

