# Supplementary material for: Fine Mapping of a Major Pleiotropic QTL Associated with Sesamin and Sesamolin Variation in Sesame (Sesamum indicum L.)
Source: Plants (Basel). 2021 Jun 30;10(7):1343. doi: 10.3390/plants10071343 (PMC8309374; doi:10.3390/plants10071343)
Supplement: Supplementary file 1 [file plants-10-01343-s001.zip › plants-1219026-supplementary.pdf]

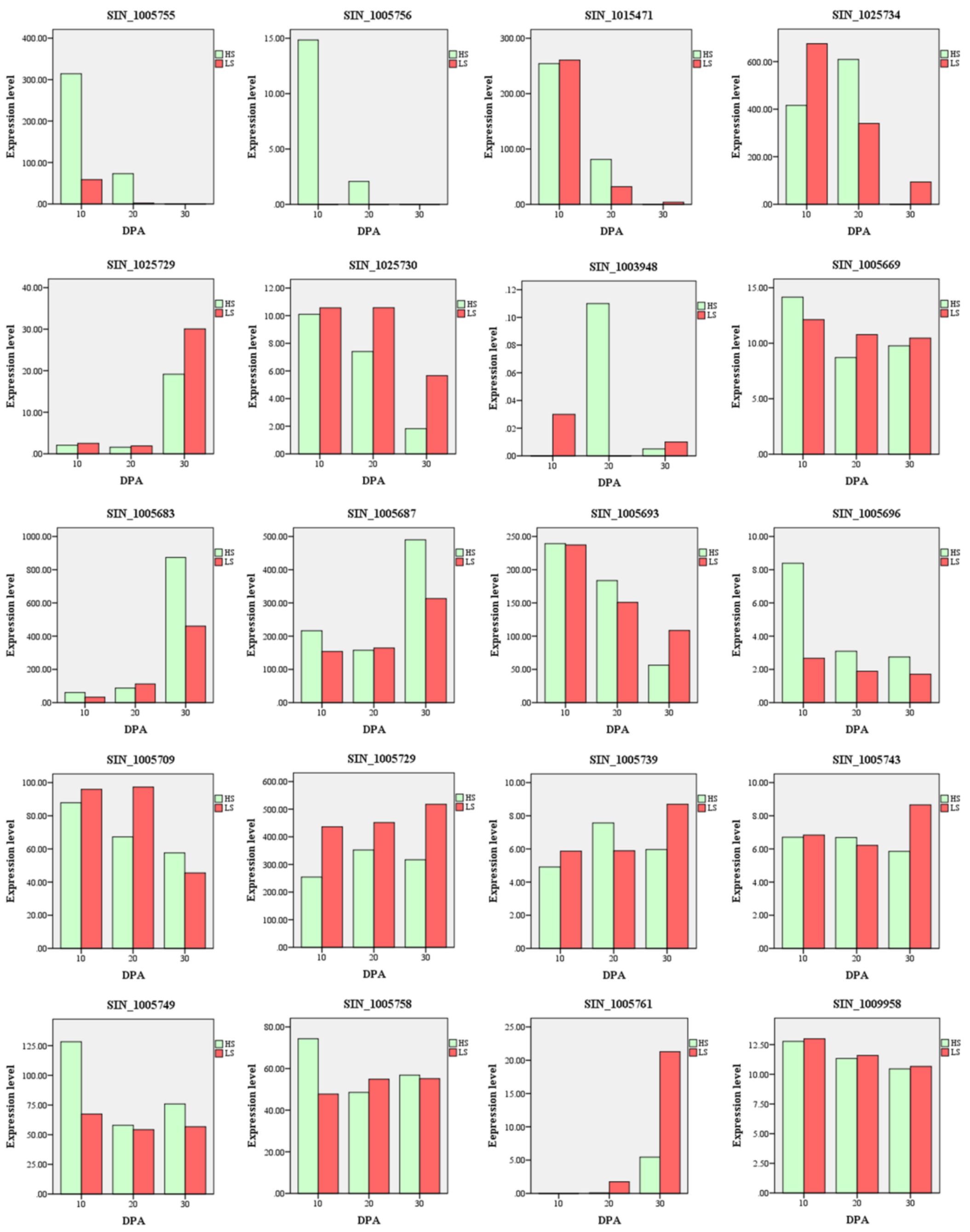

Figure S1. The expression profile of 20 genes, including the candidate regulatory genes in Zhongzhi No.13 and ZZM2748 at three stages of seed development.

Table S1. Primer sequence used for qPCR and functional annotation of the candidate regulatory genes.

Genes	Forward primer	Reverse primer	Annotation
SIN_1005755	TACCGAACCTGCACGATAGC	ATCCAGGGTCTGCGATCTCT	NAC domain-containing protein
SIN_1005756	CTCCGATCGAAAATGGTCGC	TTGATCACGAGCAGCCTCTA	Novel gene
SIN_1004293	GTTGGTCTCTTTGAGGAC	CAGCTGGATGTCTTTTGG	Reference gene
